# Carbohydrate antigens in nipple aspirate fluid predict the presence of atypia and cancer in women requiring diagnostic breast biopsy

**DOI:** 10.1186/1471-2407-10-519

**Published:** 2010-10-01

**Authors:** Susan L Deutscher, Marie Dickerson, Gerald Gui, Jessica Newton, Jeffrey E Holm, Nancy Vogeltanz-Holm, Beth Kliethermes, John E Hewett, Senthil R Kumar, Thomas P Quinn, Edward R Sauter

**Affiliations:** 1 University of Missouri Departments of Biochemistry, One Hospital Drive, Columbia, MO 65212, USA; 2 University of Missouri, Department of Biostatistics, One Hospital Drive, Columbia, MO 65212, USA; 3 The Harry S. Truman Veteran's Administration, 800 Hospital Drive, Columbia MO 65201, USA; 4 Royal Marsden NHS Foundation Trust, Fulham Road, London SW3 6JJ, UK; 5 University of North Dakota, Center for Health Promotion and Prevention Research 501 N. Columbia Rd., Grand Forks, ND 58202, USA; 6 University of North Dakota, Department of Surgery, 501 N. Columbia Rd., Grand Forks, ND 58202, USA

## Abstract

**Background:**

The goal of this prospective study was to determine (a) concentrations of the carbohydrate biomarkers Thomsen Friedenreich (TF) antigen and its precursor, Tn antigen, in nipple discharge (ND) collected from women requiring biopsy because of a suspicious breast lesion; and (b) if concentration levels predicted pathologic diagnosis.

**Methods:**

Adult women requiring biopsy to exclude breast cancer were enrolled and ND obtained. The samples from 124 women were analyzed using an anti-TF and anti-Tn monoclonal antibodies in direct immunoassay.

**Results:**

The highest median concentration in ND for TF and Tn was in women with ductal carcinoma *in situ *(DCIS). TF was higher in women with 1) cancer (DCIS or invasive) vs. either no cancer (atypia or benign pathology, p = .048), or benign pathology (p = .018); and 2) abnormal (atypia or cancer) versus benign pathology (p = .016); and was more predictive of atypia or cancer in post- compared to premenopausal women. Tn was not predictive of disease. High TF concentration and age were independent predictors of disease, correctly classifying either cancer or abnormal vs. benign pathology 83% of the time in postmenopausal women.

**Conclusions:**

TF concentrations in ND were higher in women with precancer and cancer compared to women with benign disease, and TF was an independent predictor of breast atypia and cancer. TF may prove useful in early breast cancer detection.

## Background

The Thomsen-Friedenreich (TF; Galactose-β-(1→3)-N-acetyl-D-galactosamine) antigen and its biosynthetic precursor, Tn (N-acetyl-D-galactosamine), are displayed on cell-surface proteins and lipids in 70% to 90% of adenocarcinomas including those of the breast, prostate, and ovary [1]. Antibodies to TF and Tn have been used clinically as indicators of cancer and have been detected in primary breast cancer, lymph node, and metastatic tissue samples [2,3]. Obtaining tissue samples requires invasive biopsy procedures, which result in expense and considerable discomfort to the patient. An attractive alternative would be to screen a body fluid sample obtained non-invasively to determine if the organ contains cancer, thereby minimizing invasive diagnostic procedures. Nipple discharge (ND) includes both nipple aspirate fluid (NAF), which is collected using a modified breast pump, and pathologic (P)ND, which comes forth spontaneously from one but not the other breast nipple and generally harbors a benign or malignant tumor [4]. Both types of ND can be obtained non-invasively and contain concentrated secreted proteins and lipids from the breast ductal epithelium, the cells that give rise to cancer.

We hypothesized that TF and Tn antigen, displaced on proteins and lipids that arise from cancer cells and cancer cell turnover, would be present in ND. To test this hypothesis, an initial blinded screening of 50 banked NAF samples from women with breast cancer and from asymptomatic women without cancer were screened for the presence of TF and Tn antigen. The results of this preliminary study demonstrated significant differences in the expression of TF and Tn between NAF from a breast with cancer compared to asymptomatic women [5]. Based on the encouraging findings, we instituted a multicenter prospective clinical trial in women with a breast lesion requiring diagnostic biopsy to exclude cancer.

## Methods

### Patients and Sample Collection

After receiving Institutional Review Board approval from the three institutions listed below, informed consent was obtained from all subjects prior to enrollment. 166 subjects were prospectively enrolled and samples collected in Grand Forks, ND at the University of North Dakota, in Columbia, MO at the Ellis Fischel Cancer Center and in London, UK at the Royal Marsden Cancer Center. Participants were enrolled from September 2005 to February 2008. The time between sample collection and analysis ranged from 0.7 to 25.9 months, with a median of 7.3 months. A subject was classified as postmenopausal if at least one year had passed without a menstrual period or she had undergone bilateral oophorectomy prior to enrollment. Women who had undergone hysterectomy without bilateral oophorectomy were considered postmenopausal if they were over 50 years old. If follicle stimulating hormone (FSH) levels were available, a level of 34 mIU/mL or greater was used to classify women as postmenopausal. There were four women in this category. All ND samples were collected prior to surgical biopsy. All comparisons of TF and Tn concentrations with disease were based on the histopathologic findings in the clinical report.

#### ND samples

ND (1-10 μL) samples were obtained from the breast with a lesion prior to surgery. Lesions included women with 1) PND; 2) a suspicious lesion identified on imaging, be it mammogram, ultrasound or breast magnetic resonance imaging; and/or 3) a palpable lesion that was not a simple cyst. Samples were collected as described previously [6]. Briefly, after informed consent was obtained, ND fluid was aspirated using a breast pump (NAF) or collected after the participant massaged her breast (PND). Samples were collected into capillary tubes and stored at -80°C until use.

### Assessment of TF and Tn

ND samples from women with and without breast cancer were analyzed in blinded fashion. All assays were run in triplicate. It was initially thought that an indirect immunoassay would be more sensitive than a direct assay to small changes in TF and Tn concentration, so the 42 initially collected ND samples were analyzed using an indirect sandwich immunoassay. The indirect method entailed capturing TF and Tn antigen molecules in NAF on an antibody coated surface. The surface was interrogated with a biotinylated TF or Tn ligand. Ligand binding was determined by a streptavidin-alkaline phosphate color reaction. TF and Tn concentrations were inversely proportional to the reporter signal. This assay proved unreliable, so a direct assay was developed and proved more reliable. Herein we present results for the 124 ND samples that were analyzed using a direct immunoassay.

#### Reagents

All reagents were purchased from Sigma (Sigma Chemical, St. Louis, MO) unless specifically noted. The anti-TF antibody A78-G/A7 was purchased from Glycotope (Berlin, Germany) and the BRIC-111 anti-Tn antibody was purchased from Serotec (Oxford, UK). Both antibodies were raised against desialylated red blood cells of the appropriate blood group. The TF Ab is specific for both anomeric forms of the disaccharide, including related structures on the glycolipids and shows no cross-reactivity with sialylated glycophorin [7]. The Tn antibody is specific for the alpha O-linked N-acetyl-D-galactosamine. The alpha-O linkage appears to be important. Biotinylation of antibodies and lectins was performed according to published procedures [7].

#### TF and Tn Direct Immunoassay

Each NAF sample was assayed in triplicate on the same ELISA plate for TF or Tn and the mean values were reported to the statistician. Anti-TF and anti-Tn monoclonal antibodies (200 ng/well) in a 0.1 M sodium carbonate pH 9.6 buffer were applied to Nunc-immunomax 96-well plates for 4 h at 37°C. The plates were washed with Tris-buffered saline pH 8.0 containing 0.5% Tween-20 (TTBS) and blocked with 2% bovine serum albumin (BSA) in TTBS for 2 h. One-hundred microliters of diluted ND samples (1:50-1:500) in Tris-buffered saline pH 8.0 (TBS) were added to the wells. Control wells contained various dilutions of asialofetuin (ASF) or asialo-ovine submaxillary mucin (OSM) in TBS (0 ng - 2 ng/well). ASF contains TF antigen while AOSM contains exposed Tn antigen. The ND, ASF and OSM samples were incubated at 25°C for 2 h followed by a TTBS wash. Biotinylated secondary detection proteins, anti-TF antibody (0.625 μg/ml) or Tn binding Vicia villosa lectin (1 μg/mL) in 100 μL TBS, were added to the wells and incubated for 1 hr. The unbound secondary detection proteins were removed by two washes with TTBS, while the bound biotinylated secondary detection proteins were bound with a streptavidin-horse radish peroxidase conjugate (SA-HRP) (100 μL/well of a 1 μg/mL stock) for 1 hour at 25°C. Unbound SA-HRP was removed by two TTBS washes and the wells were developed with 100 μL/well of the chromogenic substrate 2, 2'-Azino-bis(3-ethylbenzothiazoline-6-sulfonic) (Sigma) detected at 405 nm. A standard curve was performed on each plate for either TF or Tn. A range of known concentrations of TF or Tn displaying proteins, asialofetuin (TF) or ovine salivary mucin (Tn), were assayed. This allowed us to compare results from plate to plate and account for variation between plates. The data were reported as absorbance (Abs) at 405 nm per microliter of ND. The overall average between batch % coefficients of variation (%CVs) for TF and Tn were 5.5 and 8.9, respectively. The within batch average %CVs were 4.3 and 5.9, respectively. The intraclass correlation coefficient (ICC) for TF is .73 (95% CI .63 - .80) and for Tn it is .68 (95% CI .58 - .77).

### Statistical analysis

Biomarker levels for TF and Tn in the ND samples were heavily skewed and not normally distributed. We therefore described and analyzed TF and Tn biomarker levels using medians and log-transformed (Log10) means to achieve normal distributions. Univariate analyses using the Mann-Whitney-Wilcoxon signed ranks test were performed to determine median biomarker levels while t-tests were calculated to determine log10 means of the biomarkers. Logistic regression analyses are based on log10 transformed data. Three logistic regression models were used to examine the effects of TF, Tn, and menopausal status (pre-vs. post-menopausal) in predicting (a) cancer vs. no cancer diagnosis; (b) cancer vs. benign diagnosis; and (c) abnormal vs. benign diagnosis. Because menopausal status was a near significant predictor in all three overall logistic models, follow-up logistic models were conducted separately for premenopausal women and for postmenopausal women, controlling for age. ROC curves were calculated based on the logistic regression results for the postmenopausal women's data, and AUC values are presented.

## Results

### Recruitment

Of 137 women, we successfully collected NAF in 111 of 124 (90%) and PND in 13/13 (100%).

Among the 124 women evaluated, 72 (58%) were postmenopausal. Ages ranged from 19-86 years, with a median age in pre- and postmenopausal women of 45 and 61, respectively. Thirty-seven postmenopausal women took hormone replacement therapy (HRT) at one point in their lives, with nine taking HRT at the time of NAF collection. HRT use did not significantly influence the ability of TF or Tn to predict either breast abnormal pathology or cancer. Eighty-three (67%) of the participants were found to have breast cancer (11 DCIS and 72 invasive), and 41 (33%) benign disease (7 AH, 28 H, and 6 normal pathology).

### Fluid Volume and Biomarker expression not different among women with vs. those without PND

ND volume ranged from 0.5-546 microliters, with a mean of 53 and median of 25 microliters. ND volume did not vary by pathologic diagnosis nor by fluid type (NAF vs. PND). We first determined if TF and Tn expression in women requiring diagnostic biopsy due to PND differed from expression in women requiring biopsy who did not have PND. Results of logistic regression analyses conducted with women with PND (n = 13) and NAF (n = 111) were equivalent to results obtained from analyses conducted with only women providing NAF. Therefore, all reported analyses include both PND and NAF samples (N = 124).

### Biomarker expression increases with the development of precancerous changes in the breast

**Figure 1:**
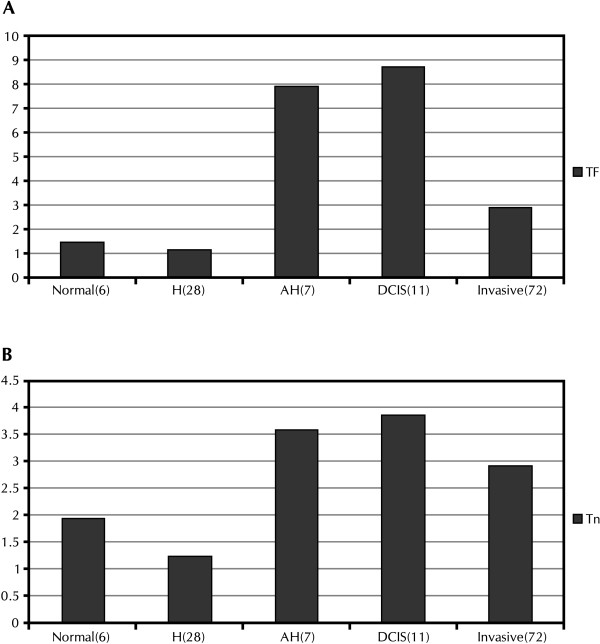
**Median values for TF (A) and Tn (B)**. H = usual hyperplasia; AH = atypical hyperplasia; DCIS: ductal carcinoma *in situ*; invasive: invasive breast cancer. Numbers in parentheses indicate the sample sizes for each group. Y axis units are Abs/μL. TF expression was higher in women with AH compared to H (p = .043), and trended higher in women with DCIS compared to normals (p = .07) and women with H (p = .09).

Median concentrations of TF and Tn were 7 and 3 fold higher, respectively (Figure 1), in women with atypical compared to those with usual hyperplasia (H). The difference in TF expression was significant (p = .043), despite limited sample size in the atypical hyperplasia group. The highest median concentration of TF and Tn were in women with ductal carcinoma *in situ *(DCIS). TF expression trended higher in women with DCIS compared to those with usual hyperplasia (p = .09) and normals (p = .07).

### TF Concentration is associated with breast atypia and cancer

Univariate analysis indicated that TF concentration was higher in women with abnormal (atypia or cancer) than benign pathology (p = .016, Table 1). TF was also higher in women with cancer (DCIS or invasive) than in women with 1) no cancer: atypia or benign pathology (p = .048), and 2) benign pathology (p = .018). Tn concentration was not significantly associated with either abnormal pathology or cancer.

**Table 1:** Univariate Analyses of TF and Tn Expression in Women With an Abnormal (Atypia or Cancer) vs. a Benign Breast Biopsy

		Overall	Benign^1^	Abnormal^1^	P value^2^
**All Subjects (N)**		124	34	90	

TF	Log10 Mean (SD)	.65 (.54)	.46 (.48)	.72 (.54)	**.016**

	Median	1.87	1.19	4.20	**.015**

	Geometric Mean (SD)	8.55 (12.73)	5.63 (12.3)	9.65 (12.78)	.12

					

Tn	Log10 Mean (SD)	.59 (.50)	.54 (.50)	.60 (.50)	.51

	Median	2.87	1.29	3.12	.57

	Geometric Mean (SD)	6.47 (9.52)	5.77 (8.48)	6.73 (9.92)	.62

					

**Premenopausal (N)**		52	18	34	

TF	Log10 Mean (SD)	.71 (.54)	.61 (.59)	.76 (.52)	.34

	Median	4.53	1.66	7.71	.27

	Geometric Mean (SD)	9.44 (12.48)	9.6 (16.04)	9.36 (10.4)	.95

					

Tn	Log10 Mean (SD)	.58 (.46)	.48 (.49)	.64 (.45)	.24

	Median	3.25	.97	3.70	.25

	Geometric Mean (SD)	5.51 (7.33)	4.71 (7.32)	5.94 (7.4)	.57

					

**Postmenopausal (N)**		72	16	56	

TF	Log10 Mean (SD)	.60 (.53)	.29 (.21)	.69 (.56)	**.007**

	Median	1.61	1.19	2.44	**.014**

	Geometric Mean (SD)	7.91 (12.96)	1.17 (1.03)	9.83 (14.12)	**.000**

					

Tn	Log10 Mean (SD)	.59 (.53)	.61 (.52)	.58 (.54)	.88

	Median	1.94	1.47	2.44	.71

	Geometric Mean (SD)	7.16 (10.83)	6.96 (9.73)	7.21 (11.21)	.94

1: Benign = normal and hyperplasia; Abnormal = atypical hyperplasia, ductal carcinoma *in situ *and invasive cancer. TF and Tn median results are in Abs/μL.

2: P value for Log10 and geometric means are based on the t-test; P value for medians are based on the Mann-Whitney-Wilcoxon test.

### TF and Tn expression based on menopausal status

We analyzed samples based on menopausal status to determine if these markers had greater predictive ability in one or the other population of women. Univariate analyses indicated that TF was a greater predictor of breast atypia and cancer among post- than among premenopausal women. TF concentration was higher in postmenopausal women with abnormal than benign pathology (Table 1, p = .007), and in cancer (DCIS or invasive) than in women with 1) no cancer (p = .025), and 2) benign pathology (p = .007). Tn concentration was not significantly associated with abnormal pathology or cancer in either pre- or postmenopausal women.

### Logistic regression

Logistic regression was conducted involving TF, Tn, and age for the following predictive groups: cancer vs. no cancer, cancer vs. benign pathology, and abnormal vs. benign pathology (Table 2). Each of these models demonstrates that in post- but not premenopausal women, high TF (odds ratios (OR) ranging from 8.53-15.56) and increasing age are independent predictors of disease (p < .05), whereas Tn concentration was not an independent predictor of disease status.

**Table 2:** Disease Prediction Based on Groups

Group	OR^1^		
	**TF**	**Tn**	**Menopausal Status**

**All Subjects**			

AbNL vs. Benign	3.29* (1.29, 8.36)	.81 (.33, 2.0)	2.19 (.95, 5.04)

CA vs, Benign	3.25* (1.27, 8.3)	.84 (.34, 2.07)	2.23 (.96, 5.2)

CA vs. No CA	2.57* (1.10, 5.98)	.82 (.32, 1.76)	2.0 (.91, 4.39)

			

	**OR^1^**		

	**TF**	**Tn**	**Age**

**Premenopausal**			

AbNL vs. Benign	.96 (.27, 3.39)	1.70 (.37, 7.81)	1.13** (1.03, 1.24)

CA vs, Benign	.86 (.23, 3.18)	1.72 (.36, 8.16)	1.17** (1.05, 1.30)

CA vs. No CA	.83 (.24, 2.84)	1.71 (.39, 7.48)	1.15** (1.04, 1.26)

			

**Postmenopausal**			

AbNL vs. Benign	15.56** (2.39, 101.2)	.35 (.09, 1.38)	1.09* (1.01, 1.18)

CA vs, Benign	13.27** (2.08, 84.63)	.35 (.09, 1.38)	1.09* (1.02, 1.18)

CA vs. No CA	8.53** (1.78, 40.74)	.32 (.09, 1.17)	1.10** (1.02, 1.18)

**^1^**OR (odds ratios) are based on logistic regression models with log10 transformations of TF and Tn values and including menopausal (pre- vs. post) status in the overall model and age in the pre- and post-menopausal group models. 95% confidence intervals are in parentheses.

*Significant at the p ≤ .05 level; **p ≤ .01.

### ROC curves

**Figure 2:**
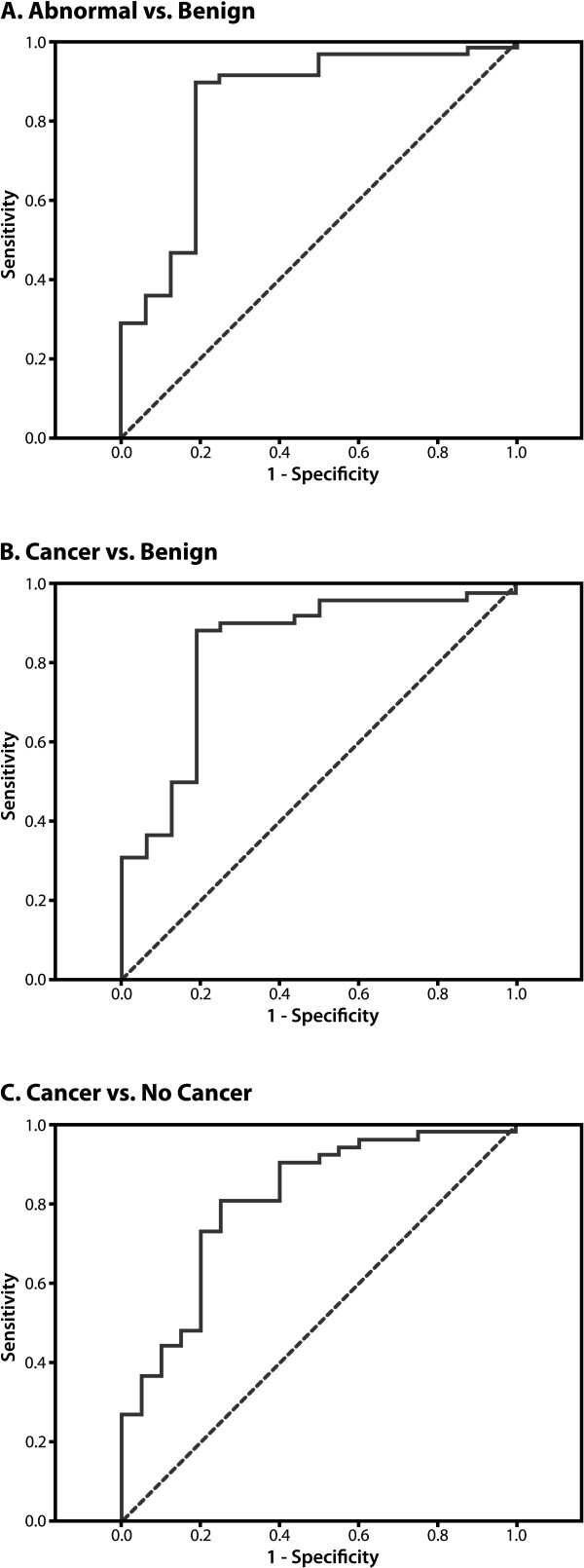
**ROC curves for the prediction of breast atypia and cancer in postmenopausal women**. TF, Tn and age information were used to generate curves comparing atypia or cancer (abnormal) vs. benign disease (AUC = 0.83) **(A)**, cancer vs. benign disease (AUC = 0.83) **(B)**, cancer vs. no cancer (AUC = 0.81) **(C)**. Sensitivity and 1-specificity are listed on the Y and × axes, respectively.

We generated ROC curves to determine how well information on TF, Tn and age predicted if a postmenopausal woman had breast atypia or cancer. Three comparisons: cancer vs. no cancer, cancer vs. benign pathology, and abnormal vs. benign pathology were conducted. The AUC values for the three comparisons were similar, ranging from .81 - .83, with the latter two comparisons having the best predictive ability (Figures 2A-C).

## Discussion

TF was the more predictive of disease in the breast than Tn. Based on our earlier published work [5], we first performed an indirect immunoassay using commercially obtained TF and Tn antibodies. The antibodies used in our initial report were no longer available, and the indirect assay using the new antibodies yielded TF and Tn concentration results that were variable and with a large number of outlier values. We next compared results using indirect and direct TF and Tn immunoassays of the same ND samples that were analyzed in our published report. We found that the direct immunoassays for TF and Tn were more reliable than the indirect assays.

We previously found that the expression of some ND cancer prediction markers varied based on whether the subject requiring surgery did or did not have PND [8]. We therefore determined if TF and Tn concentration differed based on whether the sample was PND or NAF. Since we did not identify a concentration difference, all analyses included both PND and NAF samples.

In a study involving N-nitrosomethylurea induced rat mammary cancer, Tn was found to be a biomarker of precancer in both breast tissue and serum [9]. While Tn concentrations were highest among women with DCIS and second highest among women with atypia, differences between risk groups were not significant. We cannot exclude the possibility that with a larger sample size we would have found an association.

One might ask why Tn was not associated with breast cancer, as was observed in our earlier report. First, there was a difference in the ND sample populations between our published report and current analyses. The first study was a blinded analysis performed on banked NAF samples, comparing women with breast cancer to asymptomatic women as a no cancer control. In the current analysis, all samples were obtained from women requiring surgical biopsy to evaluate an abnormal imaging study, a palpable breast mass, and/or PND. It is possible that since all women in the current study had an abnormality that required surgical biopsy, this raised the background of the assay making it more difficult to distinguish cancer from benign.

TF and Tn concentrations were notably higher in women with AH compared to those with benign disease. There are two highly effective chemopreventive agents, raloxifene and tamoxifen, for the prevention of disease in women at increased breast cancer risk [10]. These agents are especially effective in the prevention of breast cancer among women who have AH. The highest TF and Tn values were observed in women with DCIS, in whom cure rates approach 100% with proper treatment [11].

Based on our prediction models, the OR that a high TF concentration predicted disease was highest (> 13.0) for detecting 1) cancer vs. benign disease, and 2) AH or cancer vs. benign disease. The models performed less well in contrasting cancer with noncancer, in which AH was included in the no cancer category. TF concentrations in ND from women with AH appear to be more similar to women with more advanced disease than in women with benign pathology. Figure 1 also suggests that a high Tn concentration is consistent with AH or more advanced disease.

The study had limitations. First, the ability of TF or Tn to determine if a woman had AH was limited by the small number of women in the study with AH. Second, since only one ND sample collection was feasible in the women who soon after sample collection had surgery on the breast, we do not know the variability in TF or Tn expression independent of disease. A study which collects sequential ND samples from women at increased breast cancer risk, including women with a biopsy demonstrating AH, may help to address these limitations.

## Conclusions

TF and Tn are unique as biomarkers in that they are carbohydrates, whereas most disease markers both in use and undergoing evaluation are proteins. The expression of TF correlated with the presence of breast atypia and cancer. A multicenter study should be conducted to confirm our findings, and to determine the usefulness of these markers in disease detection.

## Abbreviations

Abs: absorbance; AH: atypical hyperplasia; CV: coefficient of variation; DCIS: ductal carcinoma *in situ*; H: usual hyperplasia; ICC: intraclass correlation coefficient; NAF: nipple aspirate fluid; ND: nipple discharge; OR: odds ratio; P: pathologic; ROC: receiver operating characteristic

## Competing interests

ERS is a member of the Scientific Advisory Board of Atossa Genetics, Inc. He does not hold stock nor shares in the company. Atossa Genetics did not assist in the design of the study, did not finance the study, and did not review the manuscript prior to its submission.

## Authors' contributions

SLD helped design the study, supervised the TF and Tn analyses, and participated in manuscript review. MD, JN and SK conducted the TF and Tn analyses. GG assisted in participant recruitment, sample collection, and manuscript review. JH, NV-H and JH conducted statistical analyses for the manuscript. JH and NV-H also prepared figures for the manuscript. TQ helped design the study and assisted in manuscript review. ERS helped design the study, recruited participants and collected samples that were analyzed, oversaw the entire project, and wrote the manuscript. All authors read and approved the final manuscript.

## Pre-publication history

The pre-publication history for this paper can be accessed here:

http://www.biomedcentral.com/1471-2407/10/519/prepub
